# Patient-reported data informing early benefit assessment of rare diseases in Germany: A systematic review

**DOI:** 10.1186/s13561-019-0251-9

**Published:** 2019-12-12

**Authors:** Ana Babac, Kathrin Damm, J.-Matthias Graf von der Schulenburg

**Affiliations:** Center for Health Economics Research Hannover (CHERH), Leibniz University Hannover, Otto-Brenner-Straße 7, 30159 Hannover, Germany

**Keywords:** Patient perspective, patient-reported outcomes, health economic evaluation, early benefit assessment, AMNOG, Germany

## Abstract

**Background:**

Since the implementation of the Regulation on Patient Integration (2003), the Act on the Reorganization of the Pharmaceutical Market (2011), and the Patient Rights Law (2013), the inclusion of patient perspectives has been further anchored in the German early benefit assessment process. During the assessment of rare disease interventions, patient perspectives are particularly important, as clinical studies are often designed acknowledging small samples and patients suffering from severe symptoms and the chronic course of the disease. Therefore, our research question is whether patient perspectives are considered as part of early benefit assessments for rare diseases. We also strive to examine how patient perspectives are methodologically elicited and presented.

**Methods:**

Our empirical evidence comes from a systematic review of orphan drug value dossiers submitted to the German Federal Joint Committee as well as the corresponding evaluations conducted between January 1, 2011 and March 1, 2019 (*n* = 81). Data on patient perspective integration were extracted using the following patient-reported outcome subcategories: clinical patient-reported outcomes, health-related quality of life, patient preferences, and patient satisfaction.

**Results:**

The analysis demonstrates the specific relevance of patient-reported outcomes raised as part of the medical data set and presented during the early benefit assessment process. They are predominantly presented in the form of health-related quality of life data (*n* = 75%) and clinical outcomes (*n* = 49%). Preferences (*n* = 2%) and satisfaction (*n* = 1%) are still rarely presented, although the heated methodological discussion in Germany would suggest otherwise. While various methodologies for the integration of clinical outcomes and quality of life data were found, presenting data on satisfaction and preferences still lacks methodological rigor. The German Federal Joint Committee has not yet integrated these data in their decision text. Clinical outcomes and quality of life have been included in 46% and 73% of the cases, respectively.

**Conclusions:**

The underlying analysis demonstrates that there is still a relative high potential for the regular and systematic inclusion of patient perspectives within the early benefit assessment process for rare diseases. In particular, patient preferences and patient satisfaction are still rarely included suggesting the need for a clear-cut methodological foundation and incentives.

## Introduction

### The relevance of patient perspective integration in health care

In Germany, the 2003 Patient Participation Regulation [1] as well as the 2013 Patient Rights Law [2] form the most important basis for the integration of patient perspectives within the health care system. The Patient Participation Regulation, which is linked to §140f of the German Social Insurance Code Book Volume V, regulates the mandatory involvement of patient organizations in health care decision making. Within the German Federal Joint Committee, patient organizations now have the right to advise and request, but not to vote. In 2013, patients’ position was further strengthened in terms of involvement and rights [1, 2].

In the extant literature, patient-oriented health care systems are created to extend traditional health care models using patient empowerment. Therefore, important attributes of patient-oriented health care systems are identified as: high-quality information generation and transparency, patient perspective integration through customization and collaboration, as well as the integration of patient choice and responsibility. To these, predictive and preventive instruments can also be added [3]. Consequently, empirical health economics have turned towards the collection of evidence regarding patient views. Patients should be effectively integrated during the health technology assessment process, beginning with evidence generation and value measurement and concluding with recommendations and communication of results, e.g., in the context of health policy [4].

Patient perspectives can be integrated in different ways. While summarizing data contributed by patients or their representatives is most common, this approach must be differentiated from the studies carried out from the patient perspective but contributing data, for example, collected by physicians or other health care specialists [5]. As a methodologically-grounded approach, patient-reported outcomes (PRO) stand for the reports directly originating from patients [5, 6] without the involvement of a physician or other communicators [5]. For instance, Klose et al. [7] report that the terms of PRO and outcome sometimes diverge in their interpretation. Used as a medical term, an outcome indicates an end result or intervention consequence in terms of symptoms and functioning, as well as the health-related quality of life (HRQoL). However, as reported by the PRO Harmonization Group, the discussion expanded from only including HRQoL outcomes to considering any outcome based on data provided by the patient or the patient’s proxy [8]. In this study, we follow the broader interpretation of the term PRO, as provided by the PRO Harmonization group.

Since the definition of subcategories also varies broadly, we follow the nomenclature of Klose et al. [7]. This meant that clinical PRO, HRQoL, patient satisfaction, patient experiences, and patient preferences are differentiated. Within our analysis, it seemed preferable to differentiate between the clinical PRO following the traditional medical interpretation and HRQoL due to differences in mortality, morbidity, and quality of life endpoints within the process of early benefit assessment [2]. Furthermore, patient experiences were not acknowledged separately, as they are predominantly reported in the context of patient satisfaction and patient preferences. Preferences describe whether one item is favored over another [9], meaning they withhold information regarding preferable treatment options from the affected individuals. “Preference” is often used as an umbrella term. As such, a preference measurement can result in either utility or value depending on the measurement approach [10]. There may be various reasons for the consideration of patient preferences in health care, such as improved therapy uptake or efficiency of health care interventions in practice, thus facilitating patient involvement and promoting shared decision-making in medicine. Medical decisions that are consistent with patient preferences may facilitate patient acceptance [9, 11, 12]. Therefore, patient satisfaction is also considered an important component of medical intervention assessments. However, it is a rather subjective assessment of the quality of care and is often used to incorporate the patient’s perspective on the quality of care as part of medical evaluations [12]. Patient satisfaction has not yet been well defined but is generally considered to describe a subjective assessment of medical care by patients [13]. Further, the concept can contain various elements such as medical therapy, nonmedical aspects of treatment, as well as health care infrastructure [14].

In general, the PRO can be raised via qualitative, mixed, and quantitative methodologies. Within the field of clinical PROs and HRQoL generic and disease-specific instruments are differentiated by incorporating symptom-specific modules. Patient preferences can be analyzed using contingent evaluation approaches, self-explication approaches, analytic hierarchy process, conjoint analysis, standard gamble, time trade-off approaches, as well as rating scales. For the examination of patient satisfaction, various approaches exist, such as the Patient Satisfaction Questionnaire and European Project on Patient Evaluation of General Practice Care Questionnaire [7].

### Patient perspectives in the field of rare diseases

The term “rare diseases” summarizes between 5000 and 8000 different diseases that are characterized by their severity, by their genetic origin and status as life threatening, or by the chronically debilitating course of the disease. Within the European Union, a disease is called “rare” when fewer than 5 out of 10,000 people are affected. Despite major medical advances in general, a major unmet medical need has been identified within the field of rare disease, concerning diagnostic procedures and effective treatment strategies [15]. According to the German Health Ministry, in Germany alone approximately 4 million people are affected by rare diseases [16] and although this would seem to suggest that patient perspectives are particularly important within the field of rare diseases due to its heterogeneity. There is still a lack of research on the systematic inclusion of this field during health economic processes. As demonstrated by a review of 11 national strategies regarding patient engagement, the focus of political strategies rests with the involvement of patient organizations [17]. Admitting that traditional assessments fail to endorse medical technologies for rare diseases due to a lack of power, new endpoints have been explored with names such as Patient-Centered Outcome Measures [18].

### Early benefit assessment for rare diseases in Germany

Since the Act on the Reorganization of the Pharmaceutical Market (AMNOG) within the statutory health insurance law issued in December 2010 came into effect in January 2011, all manufacturers need to provide evidence of the additional benefit of pharmaceutical products claimed over an appropriate comparator. The German Federal Joint Committee then decides whether and to what extent a drug can be granted an additional benefit and their decision forms the basis of price negotiations for the statutory health care setting [19]. Typically, the Institute for Quality and Efficiency in Health Care (Institut für Qualität und Wirtschaftlichkeit im Gesundheitswesen - IQWiG) is engaged to conduct early benefit assessment, and reports that patient perspectives play a key role in their judgments. Patient perspectives are generated using a standardized questionnaire regarding what is important to patients in terms of disease and treatment strategies [20].

However, in cases of drugs used solely for the treatment of rare diseases (orphan drugs), an additional benefit is presumed by the European drug approval, authorized in accordance with EC regulation number 141/2000 on orphan drugs [21]. In such cases, evidence must only be provided concerning the extent of the additional benefit to standard therapy for patients [22]. Here, the IQWiG is not involved in the benefit assessment, but in the estimation of patient numbers (target group, incidence, prevalence, and trends) as well as treatment costs. Only when the overall annual treatment costs of an orphan drug exceed the limit of 50 million euros for the statutory health insurance must it pass through the common early benefit assessment process [23]. Finally, decision making is taken over by the Federal Joint Committee, who describe patient involvement as the inclusion of patient representatives and patient organizations during the process but provide no further description of the procedure.

### Aims of the study

To address this gap, this article examines how the integration of patient perspectives in the assessment of benefits proceeds. To this end, we examine the development of a methodologically grounded and direct approach to patient perspective integration, using the concept of patient reported outcomes, and analyzing the data submitted during early benefit assessments for rare diseases in Germany.

## Method

### Data sources

Our empirical evidence comes from the database of the German Federal Joint Committee, withholding all procedures of early benefit assessment induced by §35a Volume V of the Social Code Book starting with the implementation of the AMNOG law [23]. All procedures with a starting date of between January 1, 2011 and March 1, 2019 were considered. In a second step, we filtered for procedures with an orphan drug status and only procedures marked “completed” were finally reviewed. Due to the exceeding of the 50-million-euro boundary or an extension of the area of application, newly developed active substances can be subject to multiple assessments.

The Federal Joint Committee makes available the following information on each procedure: the “dossier” submitted by the pharmaceutical company in accordance with the modular template, “benefit assessment,” “comments procedure,” and the “resolution” comprising “decisions” as well as the corresponding “rationales” [23]. Data were extracted from the dossier, in particular module 4, the benefit assessment, as well as the decision text developed by the Federal Joint Committee.

### Strategy of analysis

To analyze the integration of patient perspectives within the data set, the PRO concept was used following the broad definition provided by Black (2003) [6] and Patrick et al. (2003) [5] linked with the nomenclature of Klose et al. (2016) [7]. Therefore, the following subcategories have been used during the underlying analysis:

- Patient-reported outcomes clinical data (clinical PROs)
- (Health-related) quality of life (HRQoL)
- Patient preferences
- Patient satisfaction.

We did not specifically analyze the presented data for adverse events as HRQoL registers adverse events as well and mortality captures the fatal adverse events. We particularly analyzed the data presented within the synopsis section of the dossiers, the benefit assessment, as well as the decision text. The respective text passages were screened particularly searching for terms such as “patient-reported outcomes,” “quality of life”, “patient preference” and “patient satisfaction”. The identified sections were than extracted and transferred to a separate Excel sheet. Quantitative and qualitative data were reviewed equally. To provide a first impression on the relevance of the reported patient perspective within the early benefit assessment of rare diseases, we also examined the extent of the requested, and later granted, additional benefit as well as the methodologies actually considered by the GBA for each PRO data category.

## Results

### Characteristics of the data sample

Our final sample contained *n* = 81 value dossiers. The first dossier was submitted on September 15, 2011 and the last on September 15, 2018. The different disease groups are shown in Table 1. A total of 51% of the dossiers within the field of rare diseases addressed oncological indications, while metabolic diseases were the second most common, at 25%.

**Table 1:** Disease groups covered by the rare diseases benefit assessment procedures

Disease groups	Number	Percentage
Diseases of the eyes	2	2%
Cardiovascular diseases	3	4%
Infectious diseases	1	1%
Diseases of the digestive system	4	5%
Diseases of the respiratory tract	2	2%
Diseases of the blood and the blood-forming tissues	2	2%
Diseases of the musculoskeletal system	2	2%
Diseases of the nervous system	3	4%
Oncological diseases	41	51%
Metabolic diseases	20	25%
Other	1	1%
Sum	=81	=100%

Benefit assessments were predominantly (*n* = 71, 88%) conducted by the Federal Joint Committee itself. The IQWiG was commissioned with the rare diseases benefit assessment in 12% (*n* = 10) of cases, mostly when drugs were cross-passing the sales limit of 50 million euros (*n* = 7). In some cases, the manufacturer applied for an additional application area (*n* = 3). It should be noted that, whereas all agents cross passing the 50 million euro limit were commissioned to the IQWiG, applications for additional application areas were also assessed by the GBA itself.

Table 2 shows the benefit scores that were applied for by the pharmaceutical companies during those processes, as well as the GBA score granted by the Federal Joint Committee. Applied and granted benefit scores matched in 19% of cases. No additional benefit was determined in 2% of cases as part of a reassessment after the trespassing of the 50 million Euro limit conducted by the IQWiG.

**Table 2:** Benefit score of the orphan drug benefit assessment processes

Data analysis	Number of events (n)	Percentage of overall data%	Number of events (n)	Percentage of overall data%
Benefit score	Applied		Granted	
Major	45	56%	10	12%
Considerable	21	26%	0	0%
Minor	4	5%	20	25%
Not quantifiable	11	14%	49	60%
No additional benefit	–	–	2	2%
Comparison				
Matching benefit scores	15	19%	–	–

In some cases, patient populations were separated. In these cases, we solely considered the highest attained score

### Analysis

Table 3 shows a summary of the analysis of patient-reported data during the early benefit assessment process for rare diseases.

**Table 3:** Submission and consideration of PROs as part of the early benefit assessment process of rare diseases

Category	Number of PRO data sets (n)	Percentage of events in relation to overall number of processes (%)
Industry – type of PRO data submitted (module 4)		
Clinical PROs	41	51%
HRQoL	61	75%
Preferences	2	2%
Satisfaction	1	1%
Industry - extent of PRO data submitted (module 4)		
No PRO data submitted	13	16%
Data on one PRO category	35	43%
Data on two PRO categories	31	38%
Data on three PRO categories	2	2%
Early benefit assessment – type of PRO data considered in synopsis		
Clinical PRO	39	48%
HRQoL	58	72%
Preferences	0	0%
Satisfaction	0	0%
Early benefit assessment – extent of PRO data submitted		
No PRO data considered	43	53%
One PRO category	63	78%
Two PRO categories	54	67%
Three PRO categories	2	2%
GBA decision – extent of PRO data considered in the decision		
No PRO data considered	31	38%
One PRO category	25	31%
Two PRO categories	25	31%
Three PRO categories	0	0%
GBA decision – type of PRO data considered in the decision		
Clinical PROs	39	48%
HRQoL	59	73%
Preferences	0	0%
Satisfaction	0	0%
Comparison between data submitted and data considered by the GBA		
Identical number of PRO categories^a^	42	50%
Diverging number of PRO categories^a^	29	40%
Clinical PROs - not considered by GBA	8	21%
Clinical PROs - added by GBA	6	15%
HRQoL - not considered by GBA	19	31%
HRQoL – added in GBA decision	2	3%
Preferences – not considered by GBA	2	100%
Satisfaction – not considered by GBA	1	100%

*GBA* German Federal Joint Committee, *HRQoL* Health Related Quality of Life, *PRO* Patient-reported Outcomes. ^a^The number does not add up to n = 81 (all regarded processes) as some manufacturers did not provide PRO data

PROs are mostly presented in the form of self-reported clinical outcomes data (*n* = 39, 48%) followed by data on HRQoL (*n* = 61, 75%). Data on patient preferences were included twice (2%) and on patient satisfaction only once (1%). In only 15% (*n* = 12) of cases, no data on PROs were submitted. Therefore, data on PROs were presented to a relatively high extent. In 85% of cases, PROs were presented from at least one PRO subcategory. The Federal Joint Committee considered clinical PRO data in 37 (46%) cases, whereas HRQoL data were included in 59 (73%) cases. Patient satisfaction and patient preferences were not included within the decision text. Another example, which was often considered as part of the GBA decision text (*n* = 13), was the EORTC (Core Quality of Life Questionnaire). The splitting of the questionnaire items into clinical PROs and QoL PROs could also be observed here.

Table 4 shows the overall number of assessment processes for each year since the implementation of the AMNOG law. In 2017, 16 processes were initiated. Twelve processes presented data on Clinical PROs, 13 processes withheld HRQoL data, and 1 process presented data on preferences. Overall, the direct integration of patient perspectives in the form of PROs has gradually increased in its absolute number, with an increased number of induced processes since the implementation of the AMNOG law in 2011. Regarding the relative percentage of PROs in relation to the number of processes included within our analysis, no clear-cut trend is observable.

**Table 4:** Development of PRO data submissions for rare diseases over time

Items	2018		2017		2016		2015		2014		2013		2012		2011	
Assessment processes	10		16		17		15		13		3		5		2	
Clinical PROs	4	40%	12	75%	11	65%	6	40%	4	31%	0	0%	0	0%	0	0%
HRQoL	6	60%	13	81%	13	76%	11	73%	10	77%	2	67%	4	80%	2	100%
Preferences	1	10%	1	6%	0	0%	0	0%	0	0%	0	0%	0	0%	0	0%
Satisfaction	0	0%	0	0%	1	6%	0	0%	0	0%	0	0%	0	0%	0	0%

Analyzing the clinical PRO and HRQoL data, it was observable that the GBA split the surveys into symptom scales (clinical PRO data listed as part of the morbidity endpoints) and HRQoL scales. An example is the oncology specific EORTC QLQ-C30. In this context, the EQ-5D VAS scale has been categorized as part of the morbidity section. However, the EQ-5D Index has been appreciated as part of the HRQoL section. Moreover, further commonly acknowledged methodologies were the childhood health questionnaires and the Brief Pain Inventory (BPI) as well as the Functional Assessment of Chronic Illness Therapy - Fatigue (FACIT-F). On the other hand, commonly acknowledged HRQoL methodologies included disease-specific FACT-questionnaires, SF-questionnaires, and the Pediatric Quality of Life inventory (PedsQL).

The heatedly discussed categories “patient satisfaction” and “patient preferences” were rarely referred to, and when they were, it was in a qualitative manner [24–26]. Quantitative methods were not used. The dossier submitted for the agent Velmanase alfa (2018) offered “patient cases” in the form of short summaries, backing clinical PROs as well as the relevance of symptoms (preferences) and overall quality of life (not naming HRQoL in this case). There was no description of the detailed qualitative research strategy. Telotristatethyl (2017) provided “semi-structured telephone-interviews” on topics such as symptom description, preferences, and patient experiences. Some structural background data were provided, but again no description of the qualitative research strategy was included. In the case of Eftrenonacog alfa (2016), during a first phase, “focus groups” were cited as well as a “structured questioning” that also considered satisfaction. Results were presented in a qualitative manner but there was no solid description of the qualitative research strategy. Patient satisfaction and patient preferences were not included at all within the GBA decision body, thus providing no incentives for further data presentation. A detailed overview of the data can be found in Table 5.

**Table 5:** Clinical and patient-reported outcomes data considered by the German Federal Joint Committee for rare diseases

			Clinical data		Patient-reported outcome data				
	Substance	Group of disease	Mortality	Morbidity	Clinical PROs	HRQoL	Preferences	Satisfaction	Benefit score
1	Tisagenlecleucel (1st application area)	Oncologic diseases	Overall survival (OS)	Progression free survival (PFS), overall response rate (ORR)	n/a	FACT-Lym, SF-36, no usable data	n/a	n/a	non-quantifiable
2	Tisagenlecleucel (2nd application area)	Oncologic diseases	OS	Complete response/remission (CR), relapse free survival (RFS), MRD-negativ-status	EQ-5D VAS, no usable data	PedsQL (no useable data)	n/a	n/a	non-quantifiable
3	Gemtuzumab Ozogamicin	Oncologic diseases	OS	RFS, event-free survival (EFS), CR, rate of stem cell transplants	n/a	n/a	n/a	n/a	non-quantifiable
4	Velmanase alfa	Metabolic diseases	deaths, no events	Serum-Oligosaccharid-concentration, Bruininks-Oseretsky Test of Motor Proficiency (BOT-2), audiological performance with pure tone audiometry (dBHL), 3-min-stair-climbing-test, FVC, FEV, 6-min-walk-test	Childhood Health Assessment Questionnaire (CHAQ), EQ-5D-5 L Index	n/a	n/a	n/a	non-quantifiable
5	Darvadstrocel	Digestive disorders	n/a	Combined remission, clinical remission, Perianal Disease Activity Index (PDAI) (partially), no reoccurrence after clinical remission, time to clinical remission, Perianal Disease Activity Index (PDAI),	Perianal Disease Activity Index (PDAI) (partially)	Inflammatory Bowel Disease Questionnaire (IBDQ)	n/a	n/a	non-quantifiable
6	Burosumab	Metabolic diseases	None	Rachitis symptoms with Rickets Severity Scale (RSS), serumphosphat, anthropometric parameters, physical resilience (6MWT), motoric abilities (Bot-2-scale), function and pain (POSNA-PODCI), Rachitis symptoms (Radiographic Global Impression of Change (RGI-C)), pain intensity (Faces Pain Scale-Revised (FPS-R))	PROMIS (function, pain, fatigue)	SF-10 (no usable data)	n/a	n/a	non-quantifiable
7	Glycerolphenylbutyrat	Metabolic diseases	None	24-h-AUC-ammoniac-concentration in the blood	n/a	SF-36, SF-15, no usable data	n/a	n/a	non-quantifiable
8	Letermovir	Metabolic diseases	OS	Clinical relevant CMV-infection, CMV-organ disease, induction of a preemptive therapy, rehospitalization (general and after CMV-reactivation), Graft-versus-Host Disease, opportunistic bacterial, viral, and fungus infections	n/a	n/a	n/a	n/a	non-quantifiable
9	Allogenic, genetic modified T-cells	Oncologic diseases	OS, median survival, 1-year survival, 10-year survival	Immune reconstruction, acute and chronic GvHD (occurrence, time to progression)	n/a	n/a	n/a	n/a	non-quantifiable
10	Brentuximab Vedotin (new application area)	Oncologic diseases	OS	PFS, ORR, hospitalization, cutane symptomatic – symptomatic domain skindex, skin changes, mSWAT-Total-Scores, complete remission (mSWAT)	EQ-5D VAS	FACT-G (quality of life), Skindex-29 (quality of life)	n/a	n/a	minor
11	Niraparib	Oncologic diseases	OS	PFS	FOSI, no usable data	no usable data	n/a	n/a	non-quantifiable
12	Cenegermin	Diseases of the eye	mortalities	Recovery of the corneal epithelium (FDA-definition), improvement of the best corrected visual acuity, progression of the lesion depth up to cornea melting, or perforation, cornea infection	EQ-5D VAS	NEI VFQ-25-score	n/a	n/a	non-quantifiable
13	Telotristatethyl	Oncologic diseases	n/a	Stool frequency, abdominal pain, compulsory stool, nausea, adequate improvement of the gastrointestinal symptoms of the carzinoid-syndrom	EORTC QLQ-C30 – diarrhea, sleeplessness	disease related worrying (EORTC QLQ-GI.NET21)	n/a	n/a	non-quantifiable
14	Midostaurin	Oncologic diseases	OS, 5-year survival	Disease free survival, CR, rate of stem cell transplants	n/a	A: n/a / B: SF-12	n/a	n/a	considerable
15	Obinutuzumab (new application area)	Oncologic diseases	OS	PFS	n/a	FACT-Lym subscale, FACT-G	n/a	n/a	non-quantifiable
16	Avelumab	Oncologic diseases	OS	PFS	EQ-5D-VAS (health state), no usable data	FACT-M, no usable data	n/a	n/a	non-quantifiable
17	Elosulfase alfa (new benefit assessment)	Metabolic diseases	n/a	Change of walk distance, change of 3MSCT, anthropometry, respiratory functioning, wheelchair use, usage of walk aids, anthropometry, respiratory functioning	MPS Health Assessment Questionnaire (MPS HAQ)	n/a	n/a	n/a	minor
18	Daratumumab (new benefit assessment, > 50 mio € limit, new application area multiple myeloma after one pretherapy)	Oncologic diseases	OS	PFS	A: EQ-5D VAS (health state), EORTC QLQ-C30 (symptom scales), B: no relevant data	A: EORTC QLQ-C30 (health state, function scales) B: no relevant data	n/a	n/a	considerable
19	Carfilzomib (new benefit assessment, > 50 mio. € limit)	Metabolic diseases	OS	PFS	EORTC QLQ-C30 (symptom scales), EORTC QLQ-MY20 (time till first deterioration)	EORTC QLQ-C30 (function scale), EORTC QLQ-MY20 (time till deterioration)	n/a	n/a	non-quantifiable
20	Inotuzumab Ozogamicin	Oncologic diseases	OS	CR, MRD-negativity rate within patients with CR/Cri, HSZT rate	EORTC QLQ-C30 (symptom scale) (time till deterioration), no usable data, EQ-5D-VAS (health state) (time till deterioration)	EORTC QLQ-C30 (quality of life, time till deterioration), no usable data	n/a	n/a	minor
21	Nusinersen	Diseases of the nervous system	OS, 5 year survival	Disease free survival, rate of complete remission, rate of stem cell transplants	n/a	No data considered	n/a	n/a	considerable
22	Cerliponase alfa	Metabolic diseases	n/a	Proportion of responder (ML-scale), decrease of the ML−/HML-scale, time to stable decrease of ≥2 points or occurrence of value 0 on ML−/HML-scale, point value change on the ML−/HML-scale, response rate ML-scale, ML-scale, MLV-scale, MLVS-scale	n/a	PedsQL (Parent report for toddlers)	n/a	n/a	non-quantifiable
23	Blinatumomab (re-evaluation after expiry of the term)	Oncologic diseases	OS	CR/CRh/CRi rate, MRD-remission rate, rate of patients with post-baseline allo-HSZT	EORTC QLQ-C30 (symptom scale, time to decrease), no usable data, ALLSS (time till decrease), no usable data	EORTC QLQ-C30 (function scale, time till deterioration), no usable data	n/a	n/a	considerable
24	Ixazomib	Oncologic diseases	OS	PFS	BPI-SF, EQ-5D-VAS	EORTC QLQ-C30, EORTC QLQ-MY20	n/a	n/a	non-quantifiable
25	Obeticholsäure	Digestive disorders	deaths, one event	Proportion of patients with ALP < 1,67 x ULN, ALP < 1,67, Bilirubin = <ULN, ALP reduction = > 15%	Pruritus (5D und VAS)	PBC-40 (quality of life)	n/a	n/a	non-quantifiable
26	Venetoclax	Oncologic diseases	OS	PFS	EQ-5D VAS, MDASI	EORTC QLQ-C30	n/a	n/a	non-quantifiable
27	Olaratumab	Oncologic diseases	OS	PFS	n/a	n/a	n/a	n/a	considerable
28	Macitentan (new benefit assessment, > 50 mio € limit)	Cardiovascular diseases	n/a	n/a	n/a	n/a	n/a	n/a	not proven
29	Ibrutinib (new application area))	Oncologic diseases	OS	PFS	EORTC QLQ-C30 (symptom scales, time to improvement/deterioration), FACIT Fatigue (time to improvement/deterioration), EQ-5D-5 L VAS (health state, time to improvement/deterioration)	EORTC QLQ-C30 (function scales, time to improvement/deterioration)	n/a	n/a	considerable
30	Teduglutid (new application area)	Digestive disorders	n/a	pE volume reduction/change, total withdrawal of pE, pE reduction	n/a	n/a	n/a	n/a	non-quantifiable
31	Tasimelteon	Diseases of the nervous system	n/a	CGI-C (health state)	UQ-dTSD (Upper Quartile Daily Total Sleep Duration), dTSD (Daily Total Sleep Time)	n/a	n/a	n/a	non-quantifiable
32	Pitolisant	Diseases of the nervous system	n/a	Epworth Sleepiness Scale (ESS), response analysis ESS, daily kataplexie-rate	frequency and severity of narcolepsy-symptoms (sleep diary), EQ-5D VAS	n/a	n/a	n/a	non-quantifiable
33	Brentuximab Vedotin (new application area)	Oncologic diseases	OS	PFS, time to first occurrence of B-symptoms (TTBS), time till onset of an allogene transplantation (TTAllo)	EQ-5D VAS	No data available	n/a	n/a	non-quantifiable
34	Carfilzomib (new application area) [annulled]	Oncologic diseases	OS	PFS	EORTC QLQ-C30 (symptom scales)	EORTC QLQ-C30 (function scales), EORTC QLQ-MY20, FACT/GOG-Ntx	n/a	n/a	minor
35	Ibrutinib (new application area)	Oncologic diseases	n/a	n/a	n/a	n/a	n/a	n/a	not proven
36	Obinutuzumab (new application area)	Oncologic diseases	OS	PFS	EQ-5D VAS	FACT-LymS, FACT-G	n/a	n/a	non-quantifiable
37	Eftrenonacog alfa	Diseases of the blood and blood-forming organs	n/a	Annualized bleeding rate	EQ-5D-Y (VAS)	Haem-A-QoL, CHO-KLAT	n/a	n/a	non-quantifiable
38	Daratumumab [annulled]	Oncologic diseases	number of deceased, median overall survival in months	PFS	n/a	No usable data	n/a	n/a	non-quantifiable
39	Albutrepenonacog alfa	Diseases of the blood and blood-forming organs	n/a	Annualized bleeding rate	n/a	Haemo-QoL	n/a	n/a	non-quantifiable
40	Migalastat	Metabolic diseases	n/a	Cardiac endpoints, cerebrovascular endpoints, mGFRiohexol, and eGFRCKD-EPI	BPI-SF (pain scale)	SF-36v2	n/a	n/a	non-quantifiable
41	Ataluren (reevaluation after date of expiration)	Musculoskeletal diseases	n/a	Walk distance 6MWT, time till persisting deterioration (10%), changes in TFT, walk distances, NSAA score, time for four stairs (up and down)	n/a	PedsQL, PODCI (Pediatric Outcomes Data Collection Instrument)	n/a	n/a	minor
42	Afamelanotid	Metabolic diseases	n/a	Sunshine exposition	Pain scale during phototoxic episodes, photoic episodes (self-reported, patient diary)	DLQI changes (overall score)	n/a	n/a	non-quantifiable
43	Ibrutinib (new benefit assessment, > 50 mio € limit)	Oncologic diseases	OS	PFS	EQ-5D VAS	FACT-G, FACT-LymS	n/a	n/a	non-quantifiable
44	Blinatumomab [annulled]	Oncologic diseases	OS	CR/CRh, MRD-response, alloHSZT eligible patients with transplantation	n/a	n/a	n/a	n/a	non-quantifiable
45	Carfilzomib [annuled]	Oncologic diseases	OS	PFS	n/a	EORTC QLQ-C30 (health state/quality of life), EORTC QLQ-MY20 (disease symptoms)	n/a	n/a	non-quantifiable
46	Ivacaftor (new application area)	Metabolic diseases	n/a	BMI change, BMI z-score change, pulmonary exacerbation, lung functioning, response analysis lung functioning	n/a	CFQ-R	n/a	n/a	minor
47	Isavuconazol	Infectious diseases	OS	Clinical response according to the data review committee	n/a	n/a	n/a	n/a	non-quantifiable
48	Asfotase alfa	Metabolic diseases	OS	Survival without invasive ventilation, anthropometric data, motoric functioning,	Pain/disability (POSNA PODCI), Pain (BPI-SF), Lower Extremity Functional Scale (LEFS)	n/a	n/a	n/a	non-quantifiable
49	Idebenon	Diseases of the eye	n/a	Changes in visual acuity, protan- and tritan-color-perception	n/a	No usable data	n/a	n/a	non-quantifiable
50	Panobinostat	Oncologic diseases	OS	PFS	n/a	EORTC-OQL-C30, QLQ-MY-20, FACT/GOG-NTX, no valid results	n/a	n/a	non-quantifiable
51	Pomalidomid (new benefit assessment, > 50 mio € limit)	Oncologic diseases	OS	PFS	EORTC QLQ-C30 (time till deterioration of symptoms), EORTC QLQ-MY20 (time till deterioration of symptoms)EORTC QLQ-C30	EORTC QLQ-C30 (time till deterioration), EORTC QLQ-MY20 (time till deterioration)	n/a	n/a	considerable
52	Sebelipase alfa	Metabolic diseases	number of survivals	Age related weight, normalization of ALT-values, LDL-C concentration	FACIT-Fatigue	Chronic Liver Disease Questionnaires (CLDQ), PedsQL	n/a	n/a	non-quantifiable
53	Lenvatinib	Oncologic diseases	OS	PFS	n/a	n/a	n/a	n/a	non-quantifiable
54	Olaparib [annulled]	Oncologic diseases	OS	PFS	n/a	Functional Assessment of Cancer Therapy-Ovarian (FACT-O)	n/a	n/a	non-quantifiable
55	Eliglustat	Metabolic diseases	n/a	Change of spleen volume in percent, morbidity	Brief Pain Inventory (BPI), Fatigue Severity Scale (FSS)Fatigue (Fatigue Severity Scale, FSS)	SF-36	n/a	n/a	non-quantifiable
56	Nintedanib (new application area)	Respiratory diseases	time till death, time till respiratory induced death	Time till first acute exacerbation, time till first adjusted acute exacerbation, annual decrease of FVC	n/a	SGRQ-(≤ −4 point)-Responder, SGRQ-changes, SGRQ-I-score (no usable data), SOBQ-score (no significant data), CASA-Q-score cough-score (no significant data)	n/a	n/a	minor
57	Ramucirumab [annulled]	Oncologic diseases	OS	PFS	EORTC QLQ-C30 (symptoms, responder analysis symptoms), EQ-5D VAS (health state)	EORTC QLQ-C30 (health related quality of life, responder analysis)	n/a	n/a	minor
58	Pasireotid (new application area)	Metabolic diseases	n/a	Biochemical controls (GH + IGF-1, GH, reduction of tumor volume (25%), improvements of symptoms	n/a	AcroQoL, no significant data	n/a	n/a	minor
59	Ataluren [annulled]	Musculoskeletal diseases	n/a	Walk distance (6MWT, MW (m)), standing up from the back position, 10 m walk, climbing 4 stairs, descending 4 stairs	n/a	Pediatric Quality of Life Inventory (PedsQL)	n/a	n/a	minor
60	Ibrutinib [annulled]	Oncologic diseases	OS	Overall response rate (IRC-assessment), PFS	n/a	No usable data	n/a	n/a	non-quantifiable
61	Alipogentiparvovec	Metabolic diseases	n/a	n/a	n/a	n/a	n/a	n/a	non-quantifiable
62	Teduglutid	Digestive disorders	n/a	Response, withdrawal of pE, shortening of pE	n/a	SBS-QoL	n/a	n/a	minor
63	Ivacaftor (new application area)	Metabolic diseases	n/a	Change of FEV, change of BMI after 8 weeks, pulmonale eyazerbation	n/a	CFQ-R (respiratory system)	n/a	n/a	minor
64	Obinutuzumab	Oncologic diseases	OS	IRC-indicated PFS	n/a	EORTC QLQ-C30 (no significant results)	n/a	n/a	non-quantifiable
65	Cabozantinib	Oncologic diseases	OS	n/a	MDASI-THY (symptom scale)	MDASI-THY (quality of life)	n/a	n/a	minor
66	Siltuximab	Oncologic diseases	OS	Tumor response (CR and PR), fading symptoms, cancellation of corticosteroid-treatment, failure of treatment	MCD-SS (symptoms), FACIT-F-(questionnaire) (Fatigue)	SF-36, Physical Component Score (PCS), Mental Component Score (MCS)	n/a	n/a	non-quantifiable
67	Elosulfase alfa [annulled]	Metabolic diseases	n/a	Walk distance changes, changes in 3MSCT	MPS Health Assessment Questionnaire (MPS HAQ)	n/a	n/a	n/a	minor
68	Cholsäure	Metabolic diseases	n/a	n/a	n/a	n/a	n/a	n/a	non-quantifiable
69	Ruxolitinib (new benefit assessment, > 50 mio € limit)	Oncologic diseases	OS	Milt volume reduction, MFSAF v2.0	MFSAF v2.1, EORTC QLQ-C30 (no usable data)	EORTC QLQ-C30 (quality of life)	n/a	n/a	considerable
70	Riociguat	Cardiovascular diseases	OS	Changes in six-minutes-walk-distance (6MWD), changes in WHO−/NYHA-functioning classes, clinical deterioration, changes in dyspnea and fatigue (borg-scale)	n/a	EQ-5d-Index, LPH-questionnaire	n/a	n/a	minor
71	Macitentan [aufgehoben]	Cardiovascular diseases	Death till EOS	Reaching first morbidity or mortality event (EOT), changes in the 6-min-walk-distance, hospitalization, changes in the Borg-dyspnea-index, improvement of the WHO/NYHA-class	n/a	SF-36	n/a	n/a	minor
72	Pomalidomid [annulled]	Oncologic diseases	OS	PFS	n/a	EORTC QLQ-C30 (No significant results in 13 of 15 subscales, physical functioning and nausea improvement), EORTC QLQ-MY20, EQ-5D (no significant results)	n/a	n/a	considerable
73	Ponatinib	Oncologic diseases	death cases, OS	Hematologic response (HR), zytogenic response (CCyR), PFS, molecular response	n/a	n/a	n/a	n/a	non-quantifiable
74	Bosutinib	Oncologic diseases	OS	Zytogenic response (CyR), molecular response, hematologic response, PFS	n/a	EQ-5D (no usable data), Fact-Leu (no usable data)	n/a	n/a	non-quantifiable
75	Brentuximab Vedotin	Oncologic diseases	OS	PFS, event-free survival, objective response rate, complete remission, decline B-symptoms, stem cell transplant rate	n/a	n/a	n/a	n/a	non-quantifiable
76	Decitabin	Oncologic diseases	OS	CR	n/a	EORTC QLQ-C30 (no valid data), EORTC QLQ-C30 (no valid data)	n/a	n/a	minor
77	Ruxolitinib [annulled]	Oncologic diseases	OS	Spleen volume reduction (MFSAF) version 2.0	EORTC QLQ-C30 (fatigue)	EORTC QLQ-C30 (quality of life), EORTC QLQ-C30, FACT-Lym (no valid data)	n/a	n/a	minor
78	Ivacaftor	Metabolic diseases	n/a	FEV (1%), BMI (inkl. Z-Wert), pulmonary exacerbation (PE)	CFQ-R (respiratory system, child and parents), EQ-5D	n/a	n/a	n/a	considerable
79	Pasireotid	Metabolic diseases	n/a	Proportion of responders, proportion of reducers, mUFC-basic value, blood pressure, LDL-cholesterol, weight	Beck-Depressions-Inventar	Cushing’s Quality of Life Questionnaires Questionnaire (CushingQoL)	n/a	n/a	minor
80	Tafamidis Meglumin	Other diseases	n/a	Neuropathy Impairment scale of the lower limb (NIS-LL), NIS-LL responder	n/a	Norfolk QOL-DN	n/a	n/a	minor
81	Pirfenidon	Respiratory diseases	n/a	n/a	n/a	n/a	n/a	n/a	non-quantifiable

Data source: German Federal Joint Committee (GBA), GBA decisions, table of study results according to the presented endpoints

## Discussion

### Summary of findings

The present study analyzes data on the direct involvement of the patient perspective, particularly in the form of PROs, which are submitted, evaluated, and considered during the early benefit assessment process for rare diseases in Germany. The results demonstrate that patient perspectives predominantly enter the process via clinical PROs and HRQoL. However, in comparison with clinical PRO and HRQoL, the categories “patient satisfaction” and “patient preferences” were rarely referred to, and if they were, it was in a qualitative manner that lacked a solid description of the methodological foundation within qualitative research. Nevertheless, we found that 16% of the orphan drug dossiers did not present any data on PROs.

### Significance in the context of literature

To our knowledge, this study provides unique insights into the inclusion of patient perspectives within the early benefit analysis process, in particular using PROs as part of the early benefit assessment of orphan drugs.

Braithwaite et al. [27] highlight again the importance of PROs in the field of rare diseases since some of the methods used in this field of research permit smaller sample sizes. They also pinpoint the importance of primary outcome measures in general and that, in particular, traditional outcome measures have failed to demonstrate efficiency. While considerable progress has been made in the development of associated measures, it is still difficult to find tools for less common indications [27]. This may be one of the explanations for low acceptance and / or submission of PROs in the field of rare diseases.

control Changes within the political framework can affect pharmaceutical companies’ submission behavior. For example, before the introduction of the Patient Rights Law in 2013 [2], only three dossiers were submitted, while afterwards, the number of dossiers for orphan drugs increased to approximately 16 per year. However, the data cannot capture the possible impacts of changes in legislation since these events occur at a small rate and could be falsified by the overall orphan drug submission rate.

Furthermore, the methodological developments within the health economic environment in Germany can also influence the development of data submission and its appraisal. In 2013, the IQWiG discussed changes to its methodology for the very first time. Institutes and industrial representatives argued for the direct, transparent, and systematic integration of patient perspectives, in particular patient preferences and the definition of the precise integration processes [24]. In 2015, another discussion of the IQWiG general methods paper was published. However, the focus of the discussion concerning patient perspectives integration was predominantly in relation to the reintegration of patient satisfaction as optional data [25]. Moreover, in 2017, the last recorded methodological discussion was published and, in this context, the systematic direct integration of patient perspectives was again demanded in several parts of the IQWiG methods paper, e.g., the clear-cut acknowledgement of patient preferences [26]. The first pilot projects concerning the measurement and inclusion of patient preferences in health economic evaluation were published in 2013 (Analytic Hierarchy Process (AHP) [28, 29] and 2014 (Conjoint Analysis) [30, 31]. Although the named projects and discussions seem to lay the basis for the methodologically grounded inclusion of patient perspectives, the first inclusions of patient preferences were recorded in 2017 and 2018 in the field of rare diseases. However, since many of the above-named quantitative methods are not appropriate in the field of rare diseases due to the limitation of small sample sizes (see also [18]) (an exception is the AHP [32, 33]), further specified requirements for qualitative data presentation are required, as well as incentives for their adaptation. The same arguments hold for patient satisfaction, which is referred to as additional submittable data but in its patient-centeredness is relevant by definition.

#### Clinical patient-reported outcomes and health related quality of life

Furthermore, in terms of particular relevance, it has been argued that the documentation of clinical PROs - for example, as part of the phenotype “pain” - offers the chance to better align treatment options and outcomes [34].

Casamayor et al. [35] analyzed whether PROs in oncology matter in health technology assessments conducted in Germany, France, and the UK, and found that an improvement in such outcomes did not increase the chance of a positive health technology assessment (HTA) recommendation. The authors also demonstrated that PROs assessing Quality of Life (51/57, 89.4%) and pain measures (18/57, 31.6%) are the most common. PROs were not mentioned at all in 35.1% of cases [35]. Although our analysis examines HRQoL measures and morbidity-focused clinical PROs, the tendencies of both research papers seem to be similar. An early stage analysis of the first 25 dossiers in Germany regarded independently of the targeted indication demonstrated that in the beginning HRQoL outcomes were not considered during the early benefit process for different reasons [36, 37]. In our analysis, we found that this category was the most acceptable for the Joint Federal Committee. However, the general position of the Federal Joint Committee on the importance of quality of life data has changed significantly in the last 10 years. Initially rated as supporting or complementary information, quality of life data is today accounted equivalent to endpoints of mortality and morbidity [38]. Nevertheless, there are methodological questions regarding measurement and distinction that are not yet clear.

#### Patient preferences

The proportion of dossiers including data on patient preferences was quite low. Obradovic and Rauland [39] state that approximately 25% of all dossiers published between 2011 and 2014 referred to some extent to patient preferences. However, the database used seems to be more broadly designed. In the case of the present study, we included data from the as by the pharmaceutical company submitted studies but this also prompts further research questions regarding the differences between the integration of patient perspectives in the field of rare diseases and other indications. Of course, many quantitative measurement methods such as choice experiments/conjoint analyses are hardly feasible in the field of rare diseases. Furthermore, their specific aim is to compare different treatment methods (trade-off), which are often not provided in the field of rare diseases.

Although we controlled for incentives to render data on patient perspectives in a direct and systematic way considering PROs, it must also be stated that there are some factors outside the set framework that may also influence the presentation of data. For example, the benefit score and associated documents form the basis for price negotiation in Germany [40, 41].

Besides, the methodological foundation for patient preferences has also been developed in an international context and substantial literature has been published. The International Society for Pharmacoeconomics and Outcomes Research (ISPOR) developed a good research practice checklist for conjoint-analysis in health.

The checklist included 10 items covering the research question, levels and attributes, task development, the design of the experiment, preference elicitation, design of instruments, data-collection, analyzing statistical data, results and conclusions as well as study presentation standards. Even though, not endorsing a specific methodological approach, the checklist can serve as a good foundation for further discussions of good research practice for the application of conjoint-analysis methods in health care studies [42]. Besides, further research efforts give in depth advice concerning specific elements of the research process, for example the experimental design [43]. Several studies review the usage of different methodologies raising patient preferences systematically for different indications such as for example diabetes [44, 45]. Further, CONSORT guidelines advise on the reporting of PRO data in general [46]. This study contributed to the existing literature by outlining the methodology of PRO data inclusion within the field of rare diseases in Germany.

### Limitations

In terms of limitations, data on clinical PROs could only be identified as such as long as they were highlighted as a self-reported measure or indicated to be a patient-reported measure. When no particular definition was provided, we assumed that the endpoint was physician-reported. We assume that almost all endpoints were specifically marked as patient-reported, as dossier providers have often argued that clinical PROs are particularly relevant to the patient and should therefore be specifically considered during the valuation and decision-making process. Furthermore, PROs are clearly defined as self-reported. However, in the case of clinical PROs, the reporting system was sometimes not indicated. In these cases, we searched for the primary classification of the symptoms scale.

In addition, pharmaceutical companies present their HRQoL data as a whole data subset. However, the Federal Joint Committee separates parts of the questionnaires selectively regarding mortality endpoints and HRQoL endpoints. Therefore, the data reveal a splitting of the datasets rendered by the pharmaceutical company. Endpoints were not shifted as this would not reflect the actual status quo of the data presented but would, rather, lead eventually to a presentation bias. However, it needs to be highlighted as a specific procedure presented by the German Federal Joint Committee and considered when selecting the appropriate data presentation technique.

Finally, it needs to be highlighted that some dossiers can fail due to formal reasons, for example not the appropriate comparator, a study population narrower than label etc. Therefore, the impact of PROs on the final decision is not always directly derivable.

## Conclusions

The underlying evaluation demonstrates that although the political basis has been strengthened and the presented concepts have been broadly laid out as part of the health economic discussion in the context of benefit analysis and cost-benefit analysis, there remains a broad potential for the development of the practical framework regarding the systematic inclusion of patient perspectives, especially in referring to patient preferences and patient satisfaction, particularly considering the example of early benefit assessments for rare diseases in Germany. In this regard, it is interesting that patient preferences are presented in a qualitative manner. The broadly discussed and exemplified (by the IQWiG) quantitative methods have not been demonstrated in the field of rare diseases to date. While methodological standards for qualitative reporting have not yet been adopted, they must be appreciated with the same thoroughness as within quantitative research settings. An according clarification of the standard guidelines needs to be demanded. Moving even one step ahead, potentials of the integration of qualitative and quantitative research may be discussed, appreciated, and scientifically monitored in this specific context. Furthermore, the interim radiation of patient satisfaction has been commented on with vehement protest. In practice, however, it is only presented in 2% of cases in the field of rare diseases, even though this topic seems highly relevant due to the predominantly chronic and severe course of diseases. Neither of the PRO categories are enlisted within the GBA decision text. Acknowledged clinical PROs are often raised by the BPI-SF (pain scale) and FACIT-F (fatigue index). On the other hand, FACT-questionnaires, SF-questionnaires, and PedsQL are often GBA-appreciated HRQoL PROs. It is noteworthy that HRQoL questionnaires are in many cases split with regard to morbidity and HRQoL items, as datasets produced by one questionnaire are submitted cohesively. In this regard, the EQ-5D VAS is often appreciated as a morbidity endpoint by the GBA and therefore, in this context, it is categorized as a clinical PRO, whereas the EQ-5D-Index is categorized as a HRQoL. Another commonly accepted example is the oncology indication specific EORTC QLQ-C30. This may lead to irritation, hindering the preparation of PRO data inclusion by pharmaceutical companies. Therefore, potential implications should be clarified.

Furthermore, the extent of PRO data presentation withholds considerable potentials. It is questionable whether morbidity-oriented clinical PROs should only be included in every second dossier, when it is highly relevant to the patient and to treatment success. Patient satisfaction and patient preferences follow by the same token. Appreciating the central role of patient perspectives within early benefit assessments and the according legal framework, the GBA decision text should particularly appreciate the consideration of patient perspectives, flagging incentives for more extensive consideration. Considering the growing financial pressure on health care systems, strengthening direct patient perspective involvement by further integrating PROs holds an immense opportunity to align health care with actual patient needs and therefore to contribute to an effective and needs-oriented health care system development.

## Data Availability

The data that support the findings of this study are available from the authors upon reasonable request.

## Abbreviations

AMNOG: Arzneimittelmarktneuordnungsgesetz - Act on the Reorganization of the Pharmaceutical Market
GBA: Gemeinsamer Bundesauschuss – Federal Joint Committee
HRQoL: Health Related Quality of life
IQWiG: Institut für Qualität und Wirtschaftlichkeit im Gesundheitswesen – Institute for Quality and Efficiency in Health Care
PRO: Patient reported outcomes
